# Functional characterization of T2D-associated SNP effects on baseline and ER stress-responsive β cell transcriptional activation

**DOI:** 10.1038/s41467-021-25514-6

**Published:** 2021-09-02

**Authors:** Shubham Khetan, Susan Kales, Romy Kursawe, Alexandria Jillette, Jacob C. Ulirsch, Steven K. Reilly, Duygu Ucar, Ryan Tewhey, Michael L. Stitzel

**Affiliations:** 1 The Jackson Laboratory for Genomic Medicine, Farmington, CT USA; 2 Department of Genetics and Genome Sciences, University of Connecticut, Farmington, CT USA; 3 The Jackson Laboratory for Mammalian Genetics, Bar Harbor, ME USA; 4 Program in Biological and Biomedical Sciences, Harvard Medical School, Boston, MA USA; 5 Broad Institute of MIT and Harvard, Cambridge, MA USA; 6 Institute of Systems Genomics, University of Connecticut, Farmington, CT USA; 7 Graduate School of Biomedical Sciences and Engineering, University of Maine, Orono, ME USA; 8 Tufts University School of Medicine, Boston, MA USA

**Keywords:** Functional genomics, Transcriptional regulatory elements

## Abstract

Genome-wide association studies (GWAS) have linked single nucleotide polymorphisms (SNPs) at >250 loci in the human genome to type 2 diabetes (T2D) risk. For each locus, identifying the functional variant(s) among multiple SNPs in high linkage disequilibrium is critical to understand molecular mechanisms underlying T2D genetic risk. Using massively parallel reporter assays (MPRA), we test the *cis*-regulatory effects of SNPs associated with T2D and altered in vivo islet chromatin accessibility in MIN6 β cells under steady state and pathophysiologic endoplasmic reticulum (ER) stress conditions. We identify 1,982/6,621 (29.9%) SNP-containing elements that activate transcription in MIN6 and 879 SNP alleles that modulate MPRA activity. Multiple T2D-associated SNPs alter the activity of short interspersed nuclear element (SINE)-containing elements that are strongly induced by ER stress. We identify 220 functional variants at 104 T2D association signals, narrowing 54 signals to a single candidate SNP. Together, this study identifies elements driving β cell steady state and ER stress-responsive transcriptional activation, nominates causal T2D SNPs, and uncovers potential roles for repetitive elements in β cell transcriptional stress response and T2D genetics.

## Abstract (web summary)

Identifying causal variants at GWAS loci is important to understand disease mechanisms. Here the authors use massively parallel reporter assays to identify type 2 diabetes-associated variants that alter cis-regulatory activity, narrowing in on the causal variants and genetic mechanisms behind the disease.

## Introduction

Type 2 diabetes (T2D) is a complex disease with both genetic and environmental risk factors that ultimately manifests when pancreatic β cells are unable to secrete adequate amounts of insulin in response to elevated blood glucose levels^1,2^. Genome-wide association studies (GWAS) have identified single nucleotide polymorphisms (SNPs) representing 403 association signals in 243 regions of the human genome (loci) for genetic risk of developing T2D^3,4^. The overwhelming majority (~90%) of these T2D-associated GWAS SNPs are non-coding, suggesting that altered transcriptional regulation is a common molecular mechanism underlying disease risk in these loci^3–6^. Identifying the functional variant(s) among multiple SNPs that are in linkage disequilibrium (LD) at each T2D GWAS locus is an important step to convert these statistical association signals into molecular and biological insights.

Although studies over the past several years clearly implicate altered islet *cis*-regulatory element (CRE) activity in T2D genetic risk and progression, they have co-localized only ~1/4 of T2D-associated loci to altered chromatin accessibility and/or gene expression levels in islets^5,7–10^. This may be partially because previous studies measured the effect of genetic variants on chromatin accessibility (caQTLs) and gene expression levels (eQTLs) in islets under steady-state conditions^7,11^, consequently missing the role of genetic variants whose functions emerge only under certain cellular conditions. Uncovering such genotype-environment interactions is critical for a complex disorder like T2D.

Endoplasmic reticulum (ER) and the unfolded protein response (UPR) contribute to physiologic processes governing β cell protein quality control, insulin processing/secretion, and to pathophysiologic events contributing to islet failure in T2D^12–15^. Mild to moderate ER stress can elicit beneficial responses, such as β cell proliferation, to meet higher demand for insulin synthesis and secretion^16^. However, sustained insulin production demands of insulin resistance, associated with modern sedentary lifestyles^17^ and overnutrition^18–20^, can intensify ER stress and activate terminal UPR, leading to β cell dysfunction and death^13,14^. Genetic modulation of β cell ER folding capacity can ameliorate^21^ or exacerbate^22^ β cell death. Non-coding T2D risk alleles may therefore modulate the transcription of genes and pathways that alter ER stress responses and the UPR.

Massively parallel reporter assays (MPRA) are functional genomic tools to interrogate the transcription activating potential of thousands of sequences simultaneously^23^. By introducing nucleotide changes in a given sequence of interest, the effect of naturally occurring variants in the human population on MPRA activity can also be elucidated^24^. Recent studies have employed MPRA to identify functional SNPs associated with different conditions including red blood cell traits^25^, adiposity^26^, osteoarthritis^27^, and eQTLs^24^.

Here, we use MPRA to comprehensively test 2512 index and genetically linked (*r*^2 ^≥ 0.8) SNP/indel alleles representing 259 GWAS association signals for T2D and related quantitative trait from the NHGRI/EBI GWAS Catalogue, as well as 4124 SNPs residing in in vivo accessible chromatin sites in human islets, for their ability to modulate transcriptional activation in β cells under steady state and (patho)physiologic ER stress conditions. We identify 1982/6621 (29.9%) SNP-containing elements that activate transcription in MIN6 and 879 MPRA activity-modulating alleles. Multiple T2D-associated SNPs alter the activity of short interspersed nuclear element (SINE)-containing elements induced by ER stress. Importantly, MPRA uncovers 220 functional variants at 104 T2D association signals, narrowing 54 signals to a single candidate SNP. By identifying elements driving β cell steady state and ER stress-responsive transcriptional activation, we nominate putative causal T2D SNPs and uncover potential roles for repetitive elements in β cell transcriptional stress response and T2D genetics.

## Results

### Selection and testing of sequences for MPRA activity in β cells

**Fig. 1:**
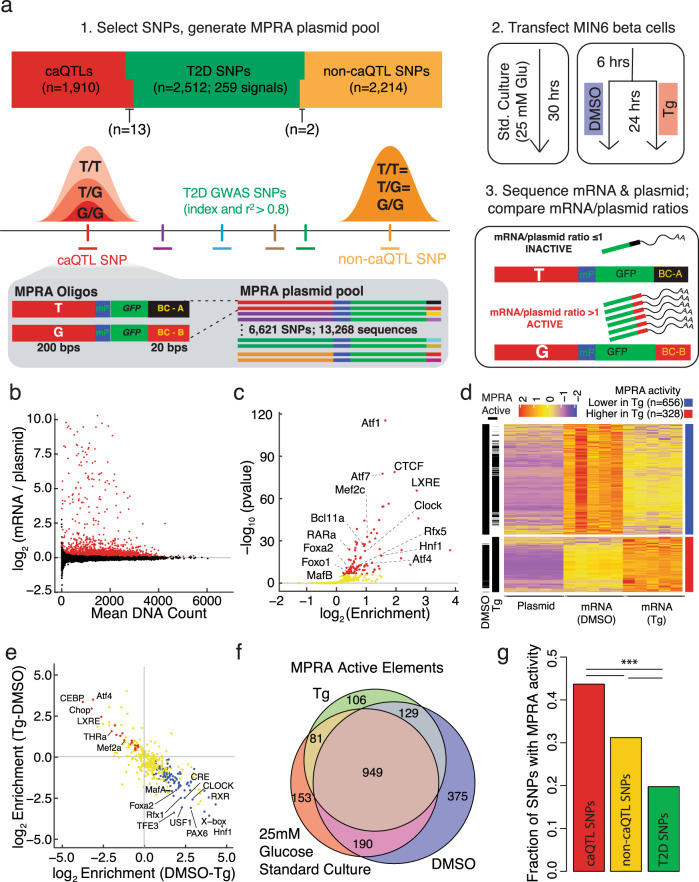
Massively parallel reporter assay (MPRA) identifies steady state and ER stress-responsive transcription activating sequences in β cells. **a** Schematic of MPRA study workflow to identify β cell transcription activating sequences. Sequences containing each allele of 2512 SNPs associated with T2D (T2D SNPs), 1910 associated with altered islet chromatin accessibility (caQTLs), and 2214 overlapping islet ATAC-seq peaks but not associated with altered in vivo islet chromatin accessibility (non-caQTL) were selected and tested for their ability to activate transcription in MIN6 β cells under three conditions. Std standard, Glu glucose; DMSO dimethylsulfoxide, Tg thapsigargin, mP minimal promoter, BC barcode. **b** Log_2_ ratios of barcode/sequence counts in *gfp* mRNA of transfected cells compared to those in the MPRA plasmid library in MIN6 β cells grown under standard culture conditions. Each point represents a 200 bp sequence that was tested. Red points denote MPRA active sequences at FDR < 1% (*n* = 2224/13,628). **c** Transcription factor (TF) binding motifs enriched in MPRA active elements (≥1 allele) in MIN6 cells grown under standard culture conditions (25 mM glucose). Red dots denote TF binding motifs enriched at FDR < 1%). **d** Heatmap of z-scores for sequences with significantly (FDR < 1%) higher (far right column, red bar; *n* = 328, mapping to 275 elements) or lower MPRA activity (blue bar; *n* = 656, mapping to 449 elements) in Tg-treated cells compared to DMSO solvent control. Black annotation bars to the left of the heatmap indicate sequences identified as MPRA active in DMSO and/or Tg based on their sequence counts in RNA vs. plasmid DNA input. **e** Transcription factor (TF) binding motifs significantly enriched (FDR < 1%) in elements identified in d with higher (red dots) or lower (blue dots) MPRA activity under ER stress. Yellow dots denote TF motifs with no significant enrichment in either comparison. **f** Venn diagram showing the number of elements (≥1 allele) with MPRA activity (FDR < 1%) in each experimental condition. **g** Fraction of 200 nucleotide sequence elements containing caQTL (*n* = 824/1910), non-caQTL (707/2218), or T2D-associated (*n* = 492/2215) SNPs with ≥1 allele identified as MPRA active in ≥1 experimental condition. Two-sided Fisher’s Exact *p* = 3.12e−16 (caQTL vs. non-caQTL SNPs), 1.50e−63 (caQTL vs. T2D SNPs), and 1.23e−19 (non-caQTL vs. T2D SNPs). ***p < 0.001.

To identify CRE sequences that activate β cell transcription and to determine how SNP alleles alter this activity, we employed MPRA in MIN6 β cells. The MPRA library consisted of two-hundred base pair (bp) sequences from the human genome containing each allele for SNPs including: (i) 2512 index or linked (EUR *r*^2 ^≥ 0.8) SNPs/indels from 259 T2D and related quantitative trait association signals in the NHGRI/EBI GWAS Catalogue^28^ (“T2D SNPs”); (ii) 1910 SNPs significantly associated with changes in human islet chromatin accessibility (“caQTL SNPs”)^7^; and (iii) 2214 SNPs that overlapped human islet ATAC-seq peaks, but were not significantly associated with changes in human islet chromatin accessibility (“non-caQTL SNPs”)^7^ (Methods; Fig. 1a).

Each sequence was cloned upstream of a minimal promoter controlling transcription of *GFP* mRNAs with distinct 20 bp barcodes in their 3′ end (Fig. 1a). This MPRA plasmid library was transfected into biological replicates of MIN6 mouse β cells and tested for transcriptional activity in three conditions: standard culture, 24-h exposure to the ER stress-inducing agent thapsigargin (250 nM Tg), or DMSO solvent control conditions (Fig. 1a). For each experimental condition (standard culture, DMSO, Tg), cells were harvested a total of thirty hours after transfection for RNA isolation, *GFP* mRNA capture, and Illumina sequencing of the sequence-associated barcodes (Methods; Fig. 1a). RNA expression of the transfected MPRA libraries was highly correlated between all replicates for each condition and clustered distinctly from the MPRA plasmid library input (Supplementary Figs. 1a–c). Under standard culture conditions, 1373 SNPs (20.7%) had at least one allele exhibiting significantly higher RNA-seq counts compared to the plasmid library input at FDR < 1% (Fig. 1b). We refer to these sequences as ‘MPRA active’ throughout the remainder of the manuscript.

While prior work has routinely used MIN6 mouse β cells to provide meaningful insights into human islet *cis*-regulatory control and the transcriptional effects of T2D risk alleles^5–7,9,11,29–31^, we first confirmed that MIN6 β cells appropriately modeled the *cis-*regulatory potential of human islets. Binding motifs of 99 transcription factors (TFs) were significantly enriched (FDR < 1%) in MPRA active elements in MIN6 β cells, which notably included motifs for TFs with reported roles in modulating beta cell identity and function (Hnf1, MafA/B, Foxo1)^32–36^, glucose-stimulated insulin secretion (Bcl11a^37^, LXRE^38^, RARa^39^), ER quality control and insulin folding and processing (Atfs), circadian regulation of β cell functions (Clock), and regulation of T2D SNP-containing *cis-*REs (Foxa2^29,40^, Rfx5^11^) (Fig. 1c and Supplementary Data 1). Several of these same motifs were enriched in sorted human islet β cell ATAC-seq peaks (e.g., Fox, Mef2c)^41^ and were among the top motifs identified as predictors of human islet regulatory features by recent computational analyses^42^. In addition, empiric binding sites (i.e., ChIP-seq peaks) for PDX1, NKX6.1, MAFB, and FOXA2 in human islets^8^ were enriched in the MPRA active elements (Supplementary Figs. 2a). In a comparison with nine human tissues, the chromatin accessibility profile of MIN6 β cells most resembled that of human islets (Supplementary Figs. 2b–c). Furthermore, human-mouse sequence similarity did not influence the probability that an element was MPRA active (Supplementary Fig. 3a), and elements overlapping human islet ATAC-seq peaks were more likely to be MPRA active in MIN6 mouse β cells than those that did not (Supplementary Fig. 3b).

Together, comprehensive testing, identification, and analysis of thousands of human sequences using MPRA in MIN6 revealed regulatory features and sequence motifs empirically linked to steady-state β cell transcriptional activation and reinforced MIN6 cells as a valid cellular model to test the β cell transcriptional potential of human sequences.

### Identification of ER stress-responsive β cell regulatory sequences

The high burden of insulin production and secretion makes β cells particularly susceptible to ER stress^43^, and ER stress has been implicated in the genetic etiology and pathophysiology of both monogenic^44,45^ and T2D^22^. Thapsigargin (Tg) blocks calcium transport into the ER lumen and has been widely used to induce the UPR in β cells^46–53^. To identify ER stress-responsive sequences, we compared MPRA activity of sequences in MIN6 cells treated with Tg versus DMSO solvent control (Fig. 1a).

Treatment with Tg increased the expression of ER stress response genes *Ddit3* (Chop), *Hspa5*, and *Edem1*, and reduced *Ins2* expression, confirming UPR induction in MIN6 cells (Supplementary Fig. 1d). ER stress increased the MPRA activity of 328 sequences (representing ≥1 allele(s) of 275 elements) and decreased MPRA activity of 656 sequences (≥1 allele of 449 elements) (Fig. 1d). Elements with increased MPRA activity under ER stress were enriched for motifs of TFs that mediate transcriptional responses to uncompensated ER stress and whose activity and/or abundance is increased in T2D patient islets^54^, such as ATF4 and *DDIT3/*CHOP^15,55–57^, as well as factors linked to pathophysiologic epigenetic changes in the beta cells of diet-induced obese mice (Mef2a^58^) and beta-cell senescence (THRa^59^) (Fig. 1e; Supplementary Data 2). Elements with decreased MPRA activity were enriched for motifs of β cell TFs that control insulin transcription and secretion^60,61^, such as MAFA, FOXA2, and PAX6 (Fig. 1e; Supplementary Data 2). Consistently, *Ins2* expression was significantly decreased by Tg (Supplementary Fig. 1d), suggesting ER stress leads to the inactivation of the β cell-specific TFs. MPRA thus revealed β cell regulatory elements that respond to ER stress and provided a functional readout of TF dynamics in this (patho)physiologic β cell stress response.

MPRA identified 1938 elements in total for which one or both alleles activated transcription in MIN6 β cells for at least one of the experimental conditions tested (Fig. 1f). T2D SNP-containing elements had the lowest proportion of active elements among the three categories of SNPs tested by MPRA (Fig. 1g; Fisher’s exact *p* = 1.50e−63, caQTL vs. T2D; *p* = 1.23e−19, non-caQTL vs. T2D), presumably because the vast majority of those tested (*n* = 2299/2512) did not overlap islet ATAC-seq peaks. Although both caQTL and non-caQTL SNPs overlapped islet ATAC-seq peaks, a significantly higher fraction of elements containing caQTL SNPs were MPRA active (Fig. 1g; Fisher’s exact *p* = 3.12e−16, caQTL vs. non-caQTL). We hypothesize this is due to the closer proximity of caQTL SNPs to islet ATAC-seq peak summits, as SNP-to-ATAC-seq peak summit proximity was associated with increased MPRA activity (Supplementary Fig. 4).

### MPRA identifies SNPs altering β cell transcriptional activity

**Fig. 2:**
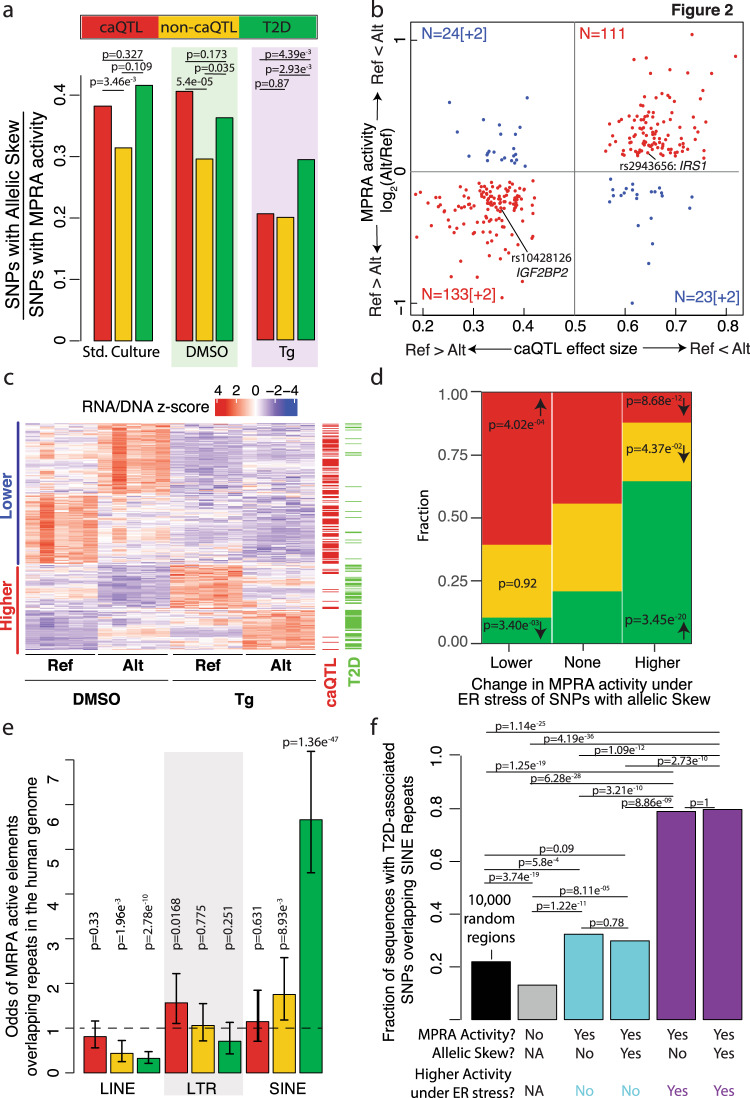
Identification of β cell transcription-modulating allele. **a** Fraction of caQTL, non-caQTL, and T2D SNP alleles that significantly alter MPRA activity in standard culture, DMSO, and Tg conditions. **b** Correlation between allelic effects on in vivo islet chromatin accessibility (*x*-axis) and MPRA activity (*y*-axis) for MPRA active elements containing islet caQTL SNPs. Quadrants 1 and 3 (red) contain SNPs where the allelic effects on chromatin accessibility and MPRA activity were concordant. The number of SNPs in each quadrant is indicated; the number of SNPs for which the relative MPRA activity lies outside the y-axis boundaries are indicated in brackets. Ref = hg19 reference allele; Alt = alternate allele. **c** Heatmap of elements containing SNPs that significantly alter MPRA activity for which overall MPRA activity is higher (red) or lower (blue) in Tg-treated vs. DMSO control-treated cells. Scale bar on top indicates z-scores obtained by centering and scaling the normalized RNA/DNA ratios for the reference and alternate alleles (5 replicates each per condition). Annotations on the right indicate whether the element (row) contains a caQTL and/or T2D-associated SNP. Note that the majority of elements with higher MPRA activity under ER stress contain T2D-associated SNPs. **d** Proportion of elements containing caQTL (red), non-caQTL (yellow), or T2D-associated (green) SNPs with allelic effects on MPRA activity (under any condition) that show lower, no change, or higher MPRA activity under ER stress conditions compared to DMSO control. A significant proportion of caQTL SNPs with allelic skew showed lower MPRA activity under ER stress, while a significant proportion of T2D-associated SNPs showed higher activity in this condition. ***p < 0.001; **p < 0.01; and *p < 0.05, Fisher’s exact test comparing elements with higher or lower MPRA activity under ER stress to those showing no change. **e** Odds of MPRA active elements (**≥**1 allele; any experimental condition) containing caQTL, non-caQTL, or T2D-associated SNPs for overlapping long interspersed nuclear element (LINE), long terminal repeat (LTR), or short interspersed nuclear element (SINE) repetitive elements. Error bars indicate the 95% confidence interval of the odds ratio estimates. **f** Fraction of T2D SNP-containing elements or 10,000 randomly selected 200 bp genomic regions (black bar) overlapping SINE repeats in the human genome. T2D SNP-containing elements are grouped based on whether they show (i) MPRA activity, (ii) allelic skew in MPRA activity, and/or (iii) higher MPRA activity under ER stress. A significantly higher fraction of T2D SNP-containing elements with higher activity under ER stress overlap SINEs compared to those without higher activity under ER stress. ***p < 0.001; **p < 0.01; and *p < 0.05 using two-sided Fisher’s exact test (corrected for multiple testing) in panels (**a**), (**d**), (**e**), and (**f**).

To identify SNPs that altered transcriptional activation in β cells, we compared MPRA activity of each allele under each experimental condition (standard culture, DMSO, Tg). Combining all three categories (caQTL, T2D, and non-caQTL), 879 SNPs exhibited allelic effects on MPRA activity (FDR < 10%) in one or more experimental condition (Fig. 2a, Supplementary Fig. 5a–c, Supplementary Data 3 and 4). For 98.2% (*n* = 332/338; Binomial test *p* = 7.2 × 10^−90^) of SNPs that altered MPRA activity in multiple conditions, the direction of allelic effects was concordant (Supplementary Fig. 5a–c). To assess if MPRA properly captured in vivo allelic effects, we assessed their concordance with caQTL effects. Strikingly, 82.8% (*n* = 246/297; *p* = 8.6 × 10^−32^, binomial test) of caQTL alleles that increased MPRA activity were associated with increased chromatin accessibility in islets (Fig. 2b), underscoring MPRA’s ability to report allelic effects relevant to their endogenous, in vivo consequences.

We next studied the impact of ER stress on SNP allelic effects and β cell transcriptional activation. As anticipated based on motif enrichment analyses (Fig. 1e), fewer caQTL SNPs exhibited significant allelic effects on MPRA activity in Tg-treated cells (Fig. 2a). This was driven by an overall reduction of activity of caQTL elements under ER stress (Fig. 2c) and associated with the enrichment of islet-specific TF binding motifs (e.g., FOXA2, MAFA) in islet caQTL ATAC-seq peaks (Fig. 1e)^7^. In contrast, a larger proportion of elements containing T2D SNPs with allelic effects by MPRA exhibited higher activity under ER stress conditions (Fig. 2c, d). Most of these T2D SNPs (*n* = 190/220) were not islet caQTL and did not overlap islet ATAC-seq peaks in our extensive set of >150,000 islet open chromatin sites from 19 donors. This may be due in part to the steady-state nature of these ATAC-seq profiles, which do not capture regulatory elements made accessible by ER stress-responsive TFs. Alternatively, the stress-responsive increases in these T2D SNP-containing elements may be mediated by repetitive elements, which have been shown to become activated by and enhance transcriptional stress responses^62–64^. Consistent with the latter, 63% (139/220) of T2D SNPs with allelic effects on MPRA activity overlapped repetitive elements. Among three repetitive element categories, only SINEs were enriched in MPRA active elements. SINEs containing T2D-associated SNPs were five times more likely to be active than any other SNP-containing repetitive elements tested (Fig. 2e). Therefore, we next asked whether T2D SNP-containing elements that overlapped SINEs showed higher activity under ER stress. Indeed, 79.4% of T2D SNP-containing elements with higher MPRA activity under ER stress overlapped SINEs, a significantly higher proportion compared to T2D SNP-containing elements with lower or no change in MPRA activity under ER stress (Fig. 2f; Supplementary Data 3). *Alu* elements, the most common SINEs in the human genome, are primate-specific^65^. When we investigated conservation in 20 mammalian genomes, T2D SNP-containing elements with higher MPRA activity under ER stress were indeed less likely to be conserved beyond the primate lineage (Supplementary Fig. 6).

In summary, MPRA revealed 879 SNPs that alter β cell transcriptional activation, including 220 T2D-associated SNPs. These SNP-containing elements exhibited distinct patterns of transcriptional activity in Tg- vs. DMSO-treated β cells, with caQTL and T2D SNP- containing elements losing or gaining activity under ER stress, respectively.

### MPRA nominates functional SNPs at T2D GWAS signals

**Fig. 3:**
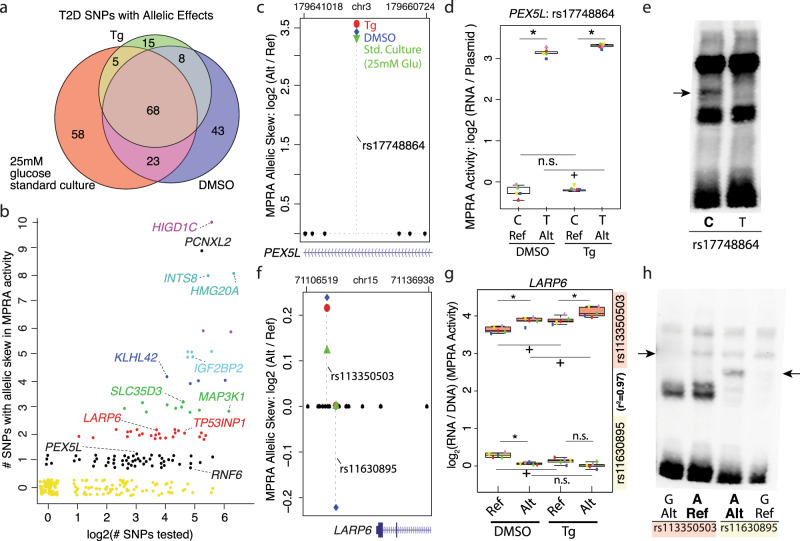
MPRA identifies functional SNPs at 104 T2D-associated GWAS signals. **a** Venn diagram showing the number of T2D-associated SNP alleles altering MPRA activity (FDR < 10%) in each experimental condition. Tg thapsigargin, DMSO dimethyl sulfoxide. **b** Scatter plot of 220/2515 T2D-associated index and high LD (*r*^2 ^≥ 0.8) SNP alleles, representing 104/259 association signals from the NHGRI/EBI GWAS catalog (*x*-axis; log scale), that significantly altered MPRA activity at FDR < 10%. Jitter in the plot is used to visually separate individual points. **c** Among eight SNPs in high LD at the *PEX5L* locus, only rs17748864 exhibited significant allelic effects on MPRA activity. Higher MPRA activity was detected for the rs17748864-T allele in all three experimental conditions compared to rs17748864-C. **d** MPRA activity for rs17748864 reference ‘C’ and alternate ‘T’ alleles in DMSO and Tg conditions. Each of the five paired biological replicates for each MPRA experiment are indicated by distinct shapes and colors. *, FDR < 10% for allelic effects on MPRA activity; +, FDR < 1% for increased MPRA activity; n.s., not significant. **e** Allele-specific binding of sequences containing rs17748864 alleles by MIN6 β cell nuclear factors. EMSA was completed with biotin-labeled probes containing the rs17748864 ‘C’ or ‘T’ allele incubated with MIN6 nuclear extract. Arrow indicates the allele-specific difference in nuclear factor binding. **f** Among 19 SNPs in high LD in the *LARP6* locus, two exhibited significant allelic effects on MPRA activity (rs113350503, rs11630895). rs113350503 alternate ‘G’ allele increased MPRA activity compared to the reference ‘A’ allele in all experimental conditions. rs11630895 reference ‘G’ allele conferred higher MPRA than the alternate ‘A’ allele in DMSO control. **g** MPRA activity for the rs113350503 reference ‘A’ and alternate ‘G’ alleles (top) and for the reference ‘G’ and alternate ‘A’ alleles of rs11630895 (bottom). Distinct data point shapes and colors of indicate each of five paired biological replicates for each MPRA experiment. *, +, and n.s. assignments are as in panel (**d**). **h** Allele-specific binding of sequences containing different rs113350503 or rs11630895 alleles by β cell nuclear factors. EMSA was completed with biotin-labeled probes containing reference or alternate alleles for each SNP and incubated with MIN6 nuclear extract. Arrows indicate allele-specific differences in nuclear factor binding. Box plots in panels (**d**) and (**g**) display the minimum, maximum, median, first quartile, and third quartile of each data set.

We next sought to nominate T2D causal variants within each locus from the set of 2512 SNPs tested by MPRA. We identified allelic effects for 220 T2D-associated SNPs in 104 distinct association signals (Fig. 3a; Supplementary Data 5–7 and Supplementary Fig. 5d–f), only 17 of which were reported index SNPs. The number of candidate functional variants identified within the association signals ranged from one to as many as ten, suggesting that some T2D association signals might have more complex effects on multiple genes and/or regulatory elements (Fig. 3b). Importantly, 6/6 T2D risk alleles that significantly altered MPRA activity and in vivo islet chromatin accessibility (islet caQTL)^7^ did so in the same direction, and T2D SNP effects on MPRA activity were consistent with their previously reported effects on in vitro luciferase reporter activity, including rs7903146 (*TCF7L2*)^6,66^, rs1635852 (*JAZF1*)^30^, rs12189774 (*VEGFA*)^67^, rs2943656 (*IRS1*)^7^, and rs10428126 (*IGF2BP2*)^7,68^.

10.4% (*n* = 23/220) of T2D SNPs exhibited specific allelic effects on MPRA activity when treated with Tg compared to baseline conditions (Fig. 3a; Supplementary Data 5 and Supplementary Fig. 5d–f). This included rs12189774, which is predicted to alter the binding of ATF5 (Supplementary Data 7), a TF that forms complexes with PDX1 and ATF4 in stressed β cells to modulate the expression of stress response and apoptosis genes^69^. Although rs12189774 has not been empirically connected to any target gene(s) to date, it may modulate ER stress-responsive VEGFA expression based on reports that ER stress elicits XBP-1(s) and ATF4 binding to the VEGFA promoter and induces VEGFA expression in pancreatic beta cells and other cell types^70–72^. 6.8% (*n* = 15/220) of T2D SNPs altered MPRA activity exclusively in ER stressed cells compared to baseline and DMSO-treated cells (Supplementary Fig. 5d–f; Supplementary Data 5).

For 54 T2D GWAS signals, a single SNP among those tested exhibited allelic effects on MPRA activity (Fig. 3b, black dots; Supplementary Data 6). For instance, rs17748864 was the only SNP among the eight tested in the *PEX5L* locus that altered MPRA activity (Fig. 3c). In contrast to the non-risk C allele, which was inactive in both DMSO and Tg conditions, the T2D risk T allele exhibited an 11-fold greater activity in MPRA and had significantly higher MPRA activity under ER stress (Fig. 3d). These allelic differences in MPRA activity were supported biochemically in electrophoretic mobility shift assays (EMSAs), which identified allele-specific binding of the non-risk C allele by a protein or complex in MIN6 nuclear extracts (Fig. 3e, arrow). Together, these data suggest that the rs17748864 risk T allele conveys robust transcriptional activation activity to this sequence by abrogating the binding of a transcriptional repressor.

For 25 T2D-associated GWAS signals, we nominated two functional SNPs (Fig. 3b, red points; Supplementary Data 6). For example, in the *LARP6* locus, rs11630895 and rs113350503 were the only functional SNPs among the 16 tested which all resided on the same tightly linked haplotype (*r*^2^ = 0.97; Fig. 3f, g). The major allele for each of these SNPs displayed opposing directions-of-effect on MPRA activity in DMSO control conditions and were affected differently by ER stress (Fig. 3f, g). As with the *PEX5L* locus, EMSAs indicate that both transcription-lowering T2D SNP alleles are bound by distinct MIN6 nuclear factors (Fig. 3h, arrows). Thus, while our results provide conclusive support for allelic effects on in vitro binding and transcriptional output, further investigation to understand their relative contributions and directions-of-effect in their endogenous context is needed. Because oligo design and synthesis for this study occurred prior to the report of credible SNP sets for T2D-associated loci^4^, our LD-based approach to select SNPs for testing did not exhaustively and comprehensively test the functionality of all T2D credible set SNPs. However, for this and other GWAS signals, MPRA has nominated multiple high-priority candidate causal SNPs for targeted and mechanistic investigation in the future (Supplementary Data 5–7).

### Integrating MPRA data with QTL maps refines T2D causal alleles/mechanisms

**Fig. 4:**
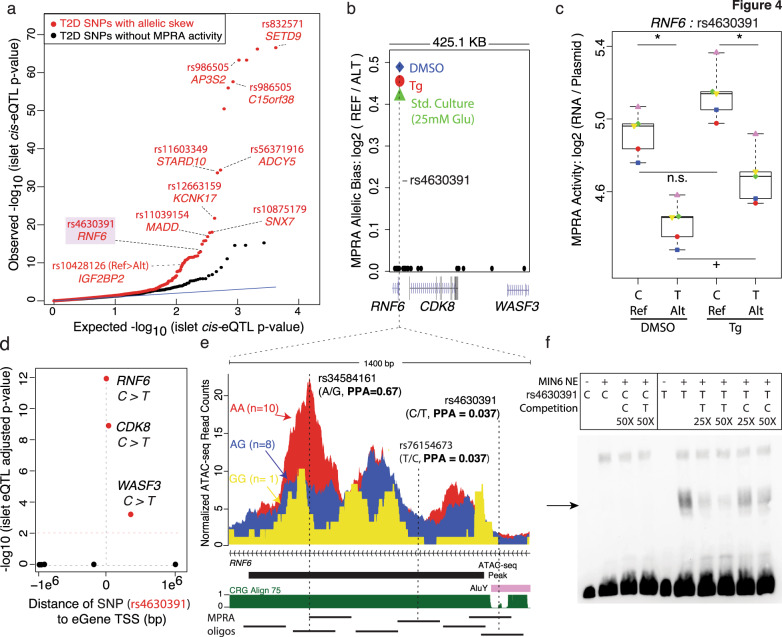
MPRA identifies putative T2D causal SNPs altering islet expression (eQTL) and chromatin accessibility (caQTL). **a** Quantile-quantile plot of observed (y-axis) vs. expected (*x*-axis) islet eQTL p-values from the InsPIRE Consortium for T2D SNPs, categorized based on whether ≥1 SNPs in LD had allelic skew (red dots), or 0 SNPs in LD had significant MPRA activity (black dots). **b** Plot of genome location of 27 SNPs in high LD (*r*^2 ^≥ 0.8) with the T2D-associated index SNP rs10507349 tested with MPRA (*RNF6* locus). Allelic effects on MPRA activity were detected for rs4630391 in the same direction across all three experimental conditions. **c** MPRA activity for the reference (‘C’) and alternate (‘T’) alleles of rs4630391 in DMSO and Tg experimental conditions. Each of the five paired biological replicates for each MPRA experiment are indicated by distinct shapes and colors. * indicates significant (FDR < 10%) allelic effects on MPRA activity; + or n.s. indicates significant (FDR < 1%) or not significant changes in MPRA activity in each condition, respectively. Box plots display the minimum, maximum, median, first quartile and third quartile of each data set. **d** Plot of InsPIRE Consortium islet eQTL association p-values between rs4630391 genotypes and expression of genes ± 1 megabase. **e** Magnified view of genomic region containing the MPRA-nominated functional T2D SNP (rs4630391) and lead islet caQTL SNP (rs34584161). Credible set SNP genetic posterior probabilities of association (PPA) are included for SNPs for which they were reported^4^. Normalized ATAC-seq reads were higher for islets from donors with rs34584161 AA homozygous genotypes (red; *n* = 10 donors) than those from AG heterozygous (blue; n = 8) or GG homozygous (yellow; *n* = 1) genotypes. ‘CRG Align 75’ indicates mappability of 75-mer sequences to the hg19 reference genome. Thick black bar indicates ATAC-seq peak in human islets. Thin black lines below indicate the MPRA oligos tested in this region. **f** Allele-specific binding of sequences containing reference and alternate alleles for rs4630391. EMSA with biotin-labeled probes containing rs4630391 C or T alleles were incubated with MIN6 nuclear extract. The arrow indicates allele-specific nuclear factor binding to the T allele, which was more efficiently competed with unlabeled T probe than unlabeled C probe.

Finally, we sought to understand the in vivo consequences of the putative functional T2D SNPs nominated by MPRA. Integration with islet caQTL revealed striking correlations between SNP effects on in vitro *MPRA a*ctivity and in vivo islet chromatin accessibility (Fig. 2b), which confirmed that the short sequence element tested by MPRA has function when residing in its broader, endogenous context. To define target genes, we determined islet transcripts whose abundance^73^ was linked to the functional T2D SNP alleles nominated by MPRA. We found that T2D functional SNPs nominated by MPRA were more enriched for islet eQTLs than T2D SNPs without MPRA activity (Fig. 4a). Integration of MPRA with caQTLs and eQTLs confirmed previously reported effects of the rs10428126 T2D risk allele, which increased reporter activity (Fig. 2b), in vivo chromatin accessibility (Fig. 2b), and *IGF2BP2* expression in islets (Fig. 4a).

Importantly, this integrated approach refined QTL maps to improve insights at additional T2D loci. For example, at the *RNF6* locus, rs4630391 was the only SNP to exert allelic effects on MPRA activity among 28 T2D SNPs in high LD that were tested (Fig. 4b). The C risk allele conveyed 30% higher MPRA activity than the T allele (Fig. 4c) and was associated with increased *RNF6*, *CDK8*, and *WASF3* expression in human islets^73^ (Fig. 4d). rs4630391 is one of eight SNPs in or immediately adjacent to an ATAC-seq peak containing an islet caQTL (Fig. 4e). Islet donors with AA genotype at the T2D-associated, lead caQTL SNP rs34584161 (*n* = 10) exhibited higher chromatin accessibility than those with AG (*n* = 8) or GG (*n* = 1) genotypes. Although rs34584161 has been recently reported as the SNP with the highest genetic posterior probability of being the causal allele for T2D association in this locus (PPAg = 0.67)^4^, only rs4630391 (PPAg = 0.037) exhibited transcription-modulating effects in MPRA (Fig. 4b). Interestingly, this SNP overlaps an Alu SINE element at the edge of an islet ATAC-seq peak and exhibits allelic effects under ER stress conditions (Fig. 4c, e). Importantly, allelic effects on MPRA activity and chromatin accessibility were concordant, i.e., the rs4630391-C allele was associated with both higher in vitro MPRA activity in MIN6 and increased in vivo chromatin accessibility in human islets (Fig. 4b, e). EMSA revealed that decreased transcriptional activity of the rs4630391-T allele was accompanied by increased T allele-specific binding in MIN6 nuclear extracts (Fig. 4f, arrow). While this does not definitively rule out that the lead caQTL SNP (rs34584161) or other SNPs in high LD are also functional, these data provide compelling functional support for rs4630391 as a putative causal variant despite its lower reported genetic posterior probability.

**Fig. 5:**
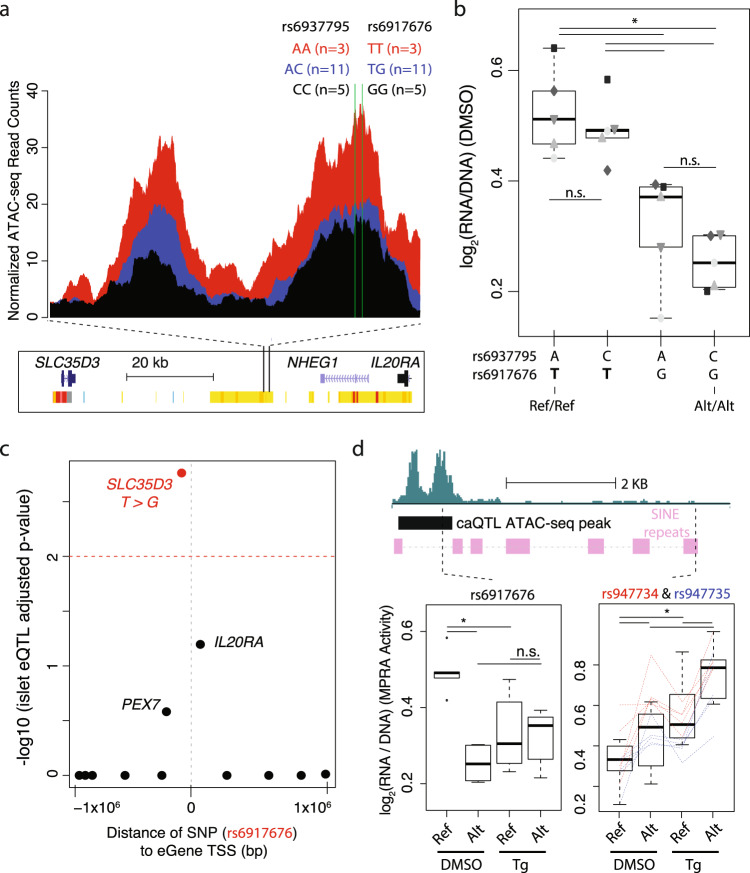
MPRA refines T2D-associated haplotype in *SLC35D3* locus. **a** Islet caQTL in the *SLC35D3* locus. Two T2D SNPs, rs6937795 and rs6917676 (15 bps apart; *r*^2^ = 0.99; green lines), are both significantly associated with altered chromatin accessibility changes (caQTLs) in the *SLC35D3* locus in islets from 19 different individuals. **b** MPRA activity (log_2_RNA/plasmid ratios) for each of the four possible rs6937795 and rs6917676 allelic combinations tested in the MPRA library. Only rs6917676 alleles altered MIN6 MPRA activity. The rs6917676 T allele is associated with both increased chromatin accessibility and MPRA activity. Shape/shading of points indicate the five paired biological replicates for each MPRA experiment. **c** Plot of InsPIRE Consortium islet eQTL -log10 association p-values between rs6917676 genotypes and expression of genes ± 1 megabase from the SNP. **d** MPRA activity (log_2_RNA/plasmid ratio) of caQTL SNP rs6917676 (left), and two additional SNPs [rs947734 (red) and rs947735 (blue); 43 bp apart] that overlap the same SINE, in ER stress (Tg-treated) compared to DMSO control conditions. Red and blue dashed lines indicate relationships between alleles for each biological replicate. * denotes FDR < 10%. Box plots in panels (**b**) and (**d**) display the minimum, maximum, median, first quartile and third quartile of each data set. Points outside the minimum and maximum values are outliers.

MPRA also refined the relative functional contributions of two T2D-associated SNPs in high LD. At the *SLC35D3* locus, we previously identified rs6937795 and rs6917676, located 15 bp apart, as islet caQTLs^7^ for which the risk alleles were associated with significantly higher chromatin accessibility in islets (Fig. 5a). Their high LD (*r*^2 ^= 0.99) make it impractical to separate the individual contributions of each SNP allele to altered regulatory function using population genetic approaches. MPRA, however, allowed each pairwise, synthetic combination to be tested independently to identify which of the SNPs has functional effects on β cell transcription. This revealed rs6917676, not rs6937795, as the SNP that altered transcriptional activity (Fig. 5b). Interrogation of recent steady-state islet eQTL data^73^ indicated that the rs6917676 T allele, which increased MPRA activity, was associated with increased *SLC35D3* expression (Fig. 5c). Allelic effects of rs6917676 were lost in ER stressed cells, while the activity and allelic effects of rs947734 and rs947735, two SNPs in high LD (*r*^2^ = 1) ~4.6 kb away in a SINE, increased (Fig. 5d). While additional mechanistic investigation is clearly needed to understand the causal relationship to T2D, this observation illustrates the value of evaluating variant haplotypes and their effects in multiple conditions.

## Discussion

In this study, we tested 13,252 sequences containing alleles of 6,621 SNPs for their ability to activate and modulate transcription in MIN6 β cells under standard culture conditions and after ER stress or paired solvent control exposures. In total, 29.9% of elements (*n* = 1982/6621) exhibited increased β cell transcriptional activity from a minimal promoter. SNP alleles in 44.3% of these elements altered MPRA activity (*n* = 879/1982), including 220 SNPs associated with T2D risk by GWAS.

Multiple lines of evidence indicate that MPRA in MIN6 mouse β cells is capable of identifying features of β cell transcription activation of human sequences and allelic effects of SNPs on this activity, despite potential limitations of cross-species testing of human sequences using episomal assays. First, MPRA active elements were enriched for both motifs and empiric binding of several islet TFs governing human islet cell identity and function under steady-state conditions. Second, under ER stress, changes in MPRA activity reflect reported in vivo changes in TF levels and their activity in β cells^54–56,58–61^. Finally, allelic effects on MPRA activity in MIN6 exhibited a significant, positive correlation with their effects on in vivo human islet chromatin accessibility. Moreover, the likelihood of MPRA activity was higher for elements in the vicinity of ATAC-seq peak summits, and SNPs closer to ATAC-seq peak summits were more likely to alter MPRA activity and chromatin accessibility. These results confirm cross-species transferability of this assay and help prioritize sequences within open chromatin regions for their importance in regulating human β cell transcriptional activity.

In total, 10.4% (*n* = 23/220) of T2D SNPs exhibiting allelic effects on MPRA activity did so only when treated with Tg compared to baseline conditions. This included a SNP that has a comparable PPAg to the top SNP in this association signal (PPAg = 0.093 vs. 0.12, respectively) and resides near *VEGFA*, a putative T2D effector gene whose expression is induced by ER stress via direct binding of the ER stress-responsive TFs XBP-1(s) and ATF4 to its promoter in pancreatic beta cells and other cell types^70–72^. 6.8% (*n* = 15/220) of T2D SNPs that altered MPRA activity were detected exclusively in ER stressed cells. This proportion of T2D SNPs with ER stress-specific effects is similar to that of the broader set of SNPs altering ER stress-specific MPRA activity in this study (7.2%). Moreover, it is comparable to the reported percentage of context-specific regulatory element use or activation^74–78^, including cytokine-responsive elements in islets (4.5%; *n* = 3798/84,162)^76^ and latent enhancers in monocytes (8.1–15%)^79^, and within the range of genetic variants reported to alter stimulus-responsive gene expression in monocytes exposed to three immunogenic stimuli (3, 9, and 17% response expression QTL upon exposures to LPS, MDP, and dsRNA, respectively)^80^. In the future, it will be interesting and important to determine if the variants altering ER stress-responsive MPRA activity alter stress-responsive islet chromatin accessibility, active histone modifications, or target gene expression in vivo and, more broadly, to test SNP effects on cis-regulatory element use or activity in response to a range of (patho)physiologic stimuli and stressors.

Surprisingly, we found that multiple T2D SNP-containing elements in SINEs were active in MPRA and responded to ER stress with increased activity. These data suggest that SINEs, and SNPs within them, may play underappreciated roles in modulating β cell transcriptional programs in response to stress or other stimuli. Recent studies have contributed to an emerging appreciation of the importance of these elements in epigenetic and transcriptional regulation, demonstrating repetitive element-mediated oncogene activation and modulation of chromatin structure^81–89^. Alu/SINEs have been shown to be transcriptionally induced by cellular stressors^63,90^ and are emerging as pervasive transcriptional modulators of cellular functions and stress^62,64,81,85,91^. Three of four SNPs tested by EMSA overlapped SINEs (rs113350503 and rs11630895 in the *LARP6* locus, rs4630391 in the *RNF6* locus) and showed specific binding for the allele with lower MPRA activity, suggesting that the activating alleles disrupt binding of a transcriptional repressor. Future studies elucidating target genes of these and other MPRA active, SINE-containing regulatory elements will be necessary to fully understand the functional consequences of sequence variation in these transcriptionally active repetitive element sequences and the potential role of SINE/Alu exaptation^83,92,93^ in the genetics of islet (dys)function and T2D.

Finally, a key challenge in T2D genetics is to identify the functional SNPs from multiple variants in high LD per association signal. This is the first study to test thousands of T2D-associated SNPs for their empiric effects on transcriptional activation in β cells. MPRA identified 220 SNP alleles associated with T2D, representing 104 distinct association signals. Candidate causal T2D SNPs nominated by MPRA include those previously studied using targeted, low throughput luciferase assays, such as rs7903146 (*TCF7L2*)^6,66^, rs1635852 (*JAZF1*)^30^, rs12189774 (*VEGFA*)^67^, rs2943656 (*IRS1*)^7^, and rs10428126 (*IGF2BP2*)^7,68^. Importantly, directions-of-effect detected for the T2D risk alleles by MPRA were consistent with those observed in each previous study. At 54 T2D association signals, only one SNP among all SNPs tested exhibited significant allelic effects on MPRA, nominating it as a putative causal SNP for that respective signal. Two or more candidate causal SNPs were identified for 50 T2D association signals, including *LARP6*, wherein EMSA and MPRA demonstrated allelic effects on both nuclear factor binding and transcriptional activity for two SNPs in high LD. The components of T2D risk at this and the 49 other GWAS signals may therefore result from a combined effect of multiple functional SNPs. Although this study was underway before T2D credible set SNPs were reported^4^, select loci illustrate how MPRA may help to evaluate and prioritize them by providing functional evidence in support of SNPs with high genetic posterior probabilities (e.g., rs7903146 (TCF7L2, PPAg = 0.59), rs3802177 (SLC30A8, PPAg = 0.57), rs10811661 and rs10811660 (CDKN2A/B, PPAgs = 0.47, 0.41), rs11603349 (CENTD2/ARAP1, PPAg = 0.22), rs4846567 (LYPLAL1, PPAg = 0.17), rs2879813 (TP53INP1, PPAg = 0.14)) or by identifying SNPs with lower genetic posterior probability for T2D association signals, such as those in the RNF6, SLC35D3, ANK1/NKX6-3, JAZF1, SPRY2, THADA, and VEGFA loci, as candidate causal SNPs. However, it is possible that SNPs with low T2D association posterior probabilities identified as functional by MPRA may not be causal, and that technical limitations such as MPRA design, fragment size, cell line used, and conditions tested, may preclude the identification of some true causal SNPs. Therefore, comprehensive testing of T2D credible set SNPs by MPRA across multiple metabolic cell types and relevant (patho)physiologic states in the near future will be critical to modify poster probabilities and nominate functional T2D variants.

## Methods

### MPRA library design

200 base pair sequences, with 100 bps flanking each side of 6621 SNPs were included in our MPRA library. The SNPs belong to three categories:

1. T2D-associated SNPs/indels: SNPs (*n* = 2299), small insertions (*n* = 72), and small deletions (*n* = 129) in linkage disequilibrium (*r*^2 ^≥ 0.8) with T2D-associated index SNPs (*n* = 259) were selected as previously described^94^ for synthesis and testing. Briefly, T2D-associated SNPs were retrieved from the NHGRI/EBI GWAS Catalog (accessed 19 January 2017) and LD-pruned using PLINK version 1.9^95^ with parameters “-maf 0.05-clump-clump-p1 0.0001-clump-p2 0.01-clump-r2 0.8-clump-kb1000” to remove index SNPs representing redundant association signals. Additional SNPs from the 1000 Genomes Phase 3 reference panel were identified and included based on their high LD (*r*^2 ^≥ 0.8) in EUR with each retained index SNP.
2. Islet chromatin accessibility quantitative trait loci (caQTLs): 1910 SNPs previously identified as having a significant association with altered in vivo chromatin accessibility in islet samples were also included^7^. Only SNPs within a given ATAC-seq peak were considered and tested for their association with altered accessibility of that peak. For 1816 caQTLs, one SNP was found to show a significant correlation with chromatin accessibility changes in human islets, all of which were included in the MPRA library (Bonferroni adjusted *p*-values < 0.023). For 94 caQTLs, two SNPs <25 bp apart showed significant correlations with islet chromatin accessibility changes, so all four allelic combinations were synthesized and tested.
3. Non-caQTL SNPs: 2214 SNPs that overlapped islet ATAC-seq peaks but did not significantly alter the accessibility of those peaks^7^ were also synthesized and tested. Since islet caQTLs were identified in a relatively small cohort of individuals (*n* = 19), the following criteria were used to include SNPs for which the caQTL study was more appropriately powered to detect associations with chromatin accessibility: (i) unadjusted *p* value >0.2; and (ii) minor allele frequency >0.125. SNPs overlapping individual-specific peaks or sharing peaks with other SNPs were removed. The 2,214 non-caQTL SNPs were randomly selected for inclusion in the MPRA library from the 15,178 SNPs that passed these inclusion criteria.

The vast majority of SNPs and elements tested belonged to only one of three categories. However, T2D-associated SNPs overlapping 13 ATAC-seq peaks were significantly associated with chromatin accessibility in islets (caQTLs). Therefore, for analysis purposes, whenever SNPs were required to belong to only one of the three categories above (such as Fig. 2a), they were not categorized as caQTLs, but as being T2D-associated only.

### MPRA library construction

The MPRA library was constructed as previously described^24^. Briefly, oligos were synthesized (Agilent Technologies) as 230 bp sequences containing 200 bp of genomic sequences and 15 bp of adaptor sequence on either end. Unique 20 bp barcodes were added by PCR along with additional constant sequence for subsequent incorporation into a backbone vector by Gibson assembly. The oligo library was expanded by electroporation into *E. coli*, and the resulting plasmid library was sequenced by Illumina 2 × 150 bp chemistry to acquire oligo-barcode pairings. The library underwent restriction digestion, and GFP with a minimal TATA promoter was inserted by Gibson assembly resulting in the 200 bp oligo sequence positioned directly upstream of the promoter and the 20 bp barcode falling in the 3′ UTR of GFP. After expansion within *E. coli* the final MPRA plasmid library was sequenced by Illumina 1 × 31 bp chemistry to acquire a baseline representation of each oligo-barcode pair within the library. Barcodes mapping to more than 1 sequence were discarded from all downstream analyses. Note: Two separate batches of the MPRA library were prepared. The first batch was used to perform MPRA under standard culture conditions. This MPRA library was then electroporated into *E. coli* to obtain a second batch of the MPRA library, which was used for the paired DMSO-Tg experiments.

### MPRA library transfection into MIN6 cells

10 million MIN6 cells were seeded in each of seven 15  cm^2^ dishes. The cells were 60–70% confluent the next day. Each 15 cm^2^ dish was replaced with 20 ml of fresh media and transfected with 7 µg of the MPRA plasmid library using 55 µl Lipofectamine 2000 (38% transfection efficiency). Six hours after transfection, media was either (i) not changed (MPRA under standard culture conditions), (ii) replaced with media containing 250 nM thapsigargin (Tg) dissolved in 0.025% DMSO, or (iii) replaced with media containing 0.025% DMSO. Thirty hours after transfection, cells were trypsinized and collected by centrifugation. Cell pellets were frozen at −80 °C. For each condition (standard culture, DMSO, or Tg), MIN6 cells were transfected on five separate days to generate biological replicates.

### RNA isolation and MPRA RNA-seq library generation

RNA was extracted from frozen cell pellets using the Qiagen RNeasy Midi kit. Following DNase treatment, a mixture of 3 GFP-specific biotinylated primers (Supplementary Data 8; #120, #123, and #126) were used to immunoprecipitated GFP transcripts using Streptavidin C1 Dynabeads (Life Technologies). Following another round of DNase treatment, cDNA was synthesized from GFP mRNA using SuperScript IV and purified with AMPure XP beads. Quantitative PCR using primers specific for GFP (Supplementary Data 8; #34 and #52) was used to determine the cycle at which linear amplification begins for each replicate. Replicates were diluted to approximately the same concentration based on the qPCR results, and PCR with primers #34 and #52 was used to amplify barcodes associated with the ~13.5k sequences included in the MPRA library for each replicate (9 cycles for standard culture, and 13 cycles for DMSO/250 nM Tg). A second round of PCR (6 cycles) was used to add Illumina sequencing adaptors to the DNA/RNA replicates. The resulting MPRA barcode libraries were spiked with 5% PhiX and sequenced using Illumina single-end 31 bp chemistry (with 8 bp index read), clustered at 80–90% maximum density.

### MPRA data analysis

Data from the MPRA was analyzed as previously described^24^. Briefly, the sum of the barcode counts for each oligo within replicates was median normalized, and oligos showing differential expression relative to the plasmid input were identified by modeling a negative binomial distribution with DESeq2^96^ and applying a false discovery rate (FDR) threshold of 1%. For sequences that displayed significant MPRA activity, a paired *t*-test was applied on the log-transformed mRNA/plasmid ratios for each experimental replicate to test whether the reference and alternate allele had similar activity. An FDR threshold of 10% was used to identify SNPs with significant effects on MPRA activity between alleles. Because the MPRA testing standard culture conditions was performed with a separate MPRA library preparation, the DMSO-Tg MPRA results were not directly compared to MPRA performed under standard culture conditions.

### Annotating repetitive elements tested with MPRA

The ‘RepeatMasker’ track for hg19 was downloaded from the UCSC genome browser. Among the ten different classes of repeats, only three classes (long interspersed nuclear element (LINE), long terminal repeat (LTR), and SINE) overlapped more than 100 elements tested with MPRA. Therefore, only these three classes of repeats were assessed for associations with MPRA activity.

### TF motif enrichment

Homer^97^ findMotifsGenome.pl script was used to investigate TF motifs enriched in a given set of elements. Elements with lower MPRA activity under ER stress were used as background to identify TF motifs enriched in elements with higher MPRA activity under ER stress, and vice-versa (parameters: hg19, -size given). 2008 T2D-associated elements with no MPRA activity were used as background to identify TF motifs enriched in the 492 T2D SNP-containing elements with significant MPRA activity (parameters: hg19, -size given). For the cross-species ATAC-seq peak analysis, ATAC-seq peaks shared with other human cell types were used as background to identify TF motifs enriched in unique ATAC-seq peaks (parameters: mm9, -size given).

### Analysis of islet ChIP-seq data

Chromatin immunoprecipitation sequencing (ChIP-seq) data from Pasquali et al.^8^ were aligned to the hg19 reference human genome as previously described^6^. Elements tested with MPRA were then overlapped with ChIP-seq peaks to conduct Fisher’s exact tests using R.

### Electrophoretic mobility shift assay (EMSA)

21-bp biotin end-labeled complementary oligonucleotides were designed with each SNP allele of interest in the 11th position of the oligo (Integrated DNA Technologies; Supplementary Data 8). For each SNP tested, complementary oligos were annealed to create double-stranded probes for each allele tested. Nuclear extract was prepared from MIN6 β cells using the NE-PER Extraction Kit (Thermo Scientific), and EMSA were completed using the LightShift Chemiluminescent EMSA kit (Thermo Scientific) according to the manufacturer’s instructions. Binding reactions consisted of 1× binding buffer, 1 µg poly dI-dC, 4 µg MIN6 nuclear extract, and 200 fmol labeled probe. Reactions were incubated at 25 °C for 25 m. For competition reactions, 25- and 50-fold excess of non-biotinylated double-stranded probes for either allele was included and pre-incubated in the reaction mixture for 15 m. DNA-protein complexes were detected by chemiluminescence. EMSAs were completed on two or more separate occasions to ensure that results were consistent.

### MPRA-based interrogation of islet eQTLs

InsPIRE Consortium^73^ islet eQTL p-values were retrieved for genes within 1 megabase (Mb) of each T2D-associated SNP included in the MPRA library. In Fig. 4a, nominal islet eQTL p-values of T2D-associated SNPs for which ≥1 SNP in high LD (*r*^2 ^> 0.8) exhibited significant allelic effects on MPRA activity were plotted and compared to those for which no SNPs in high LD exhibited MPRA activity. For locus-specific plots in Figs. 4d and 5c, nominal p-values were adjusted for multiple testing of genes within 1 Mb on either side of the SNP (Bonferroni corrected *p*-value cutoff = 0.01).

### Mapping human regulatory sequences tested with MPRA to mammalian genomes

The UCSC genome browser Liftover tool was used to map human sequences (hg19) tested with MPRA to 20 mammalian genomes (with a minimum ratio of 0.20 bases that must remap; allowing for multiple output regions). The 20 mammalian genomes are: papAnu2 (Baboon), felCat5 (Cat), PanTro6 (Chimpanzee), BosTau7 (Cow), canFAM3 (Dog), loxAfr3 (Elephant), nomLeu3 (Gibbon), gorGor3 (Gorilla), equCab2 (Horse), mm9 (mouse), ponAbe2 (Orangutan), aiMel1 (Panda), susScr11 (Pig), ochPri3 (Pika), oryCun2 (Rabbit), rn5 (Rat), rheMac8 (Rhesus), oviAri3 (Sheep), sorAra2 (Shrew), speTri2 (Squirrel). Human sequences that did not lift over to the genome assembly of a given species were subsequently classified as not conserved (with a minimum ratio of 0.20 bases that must remap; allowing for multiple output regions).

To obtain human-mouse sequence similarity measures, Liftover was performed 99 times with the minimum ratio of bases that must remap ranging from 0.01 to 1.00 in increments of 0.01 (allowing for multiple output regions). The R package ‘sm’ was used plot density of human-mouse sequence similarity and perform non-parametric bootstrap hypothesis tests of equality. Human sequences that did not liftover to the mm9 mouse genome with even 1% sequence similarity were classified as having 0% sequence similarity.

### Cross-species mapping of human ATAC-seq peaks to MIN6 ATAC-seq peaks

Human and mouse ATAC-seq data were processed as previously described^7^. Briefly, low-quality portions of reads were trimmed using Trimmomatic^98^ and aligned to the hg19 or mm9 genome assembly using Burrows Wheeler Aligner-MEM. For each replicate, duplicate reads were removed after shifting. Technical replicates were merged using SAMtools and peaks were called using MACS2^99^ (with parameters -callpeak–nomodel -f BAMPE) at FDR < 1%. ATAC-seq peak summit positions were obtained from MACS2 output files. The liftover tool in the UCSC genome browser was used to map human ATAC-seq peaks to the mouse (mm9) genome using a minimum ratio of 0.10 bases that must remap (not allowing for multiple output regions). Using bedtools, human ATAC-seq peaks mapping to the mouse genome were then overlapped with MIN6 ATAC-seq peaks.

### Identification of TF binding motifs disrupted by T2D-associated SNPs

Genes expressed in MIN6 beta cells were identified by fitting a Gaussian mixture model (two components) to RNA-seq FPKM values. After identifying genes expressed in MIN6 beta cells (FPKM values ≥ 1.63), mouse gene names were converted to corresponding human homologs using the R Package, ‘biomaRt’^100^. After filtering for expression in MIN6 beta cells, the R Package, ‘MotifBreakR’^101^, was used to identify TF binding motifs disrupted by SNPs with allelic skew in MPRA activity (Supplementary Data 4 and 7). Parameters used were: pwmList = hocomoco, threshold = 1e−2, filterp = TRUE, method = “ic”. In addition to MotifBreakR, predictions for TF binding motifs disrupted by T2D-associated and caQTL SNPs with allelic skew in MPRA activity were also obtained from SNP2TFBS^102^ (default options) (Supplementary Data 4 and 7).

### Reporting summary

Further information on research design is available in the Nature Research Reporting Summary linked to this article.

## Supplementary information

Supplementary Information

Description of Additional Supplementary Files

Supplementary Data 1

Supplementary Data 2

Supplementary Data 3

Supplementary Data 4

Supplementary Data 5

Supplementary Data 6

Supplementary Data 7

Supplementary Data 8

Reporting Summary

## Data Availability

All datasets generated and analyzed during the current study are publicly available in GEO under Accession GSE145643 (https://www.ncbi.nlm.nih.gov/geo/query/acc.cgi?acc = GSE145643), which includes fastq files containing barcode sequences in the 3′UTR of gfp in the plasmid library and MIN6 RNA samples and processed files containing the sum of all barcode counts for each test construct in the plasmid DNA and MIN6 RNA samples. Human islet ATAC-seq data were obtained from NCBI Sequence Read Archive Accession SRP117935. Summary InsPIRE Consortium73 islet eQTL statistics were obtained from https://zenodo.org/record/3408356 and SNP2TFBS predictions for transcription factor motifs altered by MPRA-modulating SNP alleles were obtained from https://ccg.epfl.ch/snp2tfbs/.

## Code availability

Code used for analysis in this paper is available at https://github.com/UcarLab/MPRA_Khetan and linked to Zenodo at 10.5281/zenodo.4974390
