# Supplementary material for: Functional characterization of T2D-associated SNP effects on baseline and ER stress-responsive β cell transcriptional activation
Source: Nat Commun. 2021 Sep 2;12:5242. doi: 10.1038/s41467-021-25514-6 (PMC8413311; doi:10.1038/s41467-021-25514-6)
Supplement: Supplementary file 1 — Supplementary Information [file 41467_2021_25514_MOESM1_ESM.pdf]

Supplementary Figures

Supplementary Figure 1

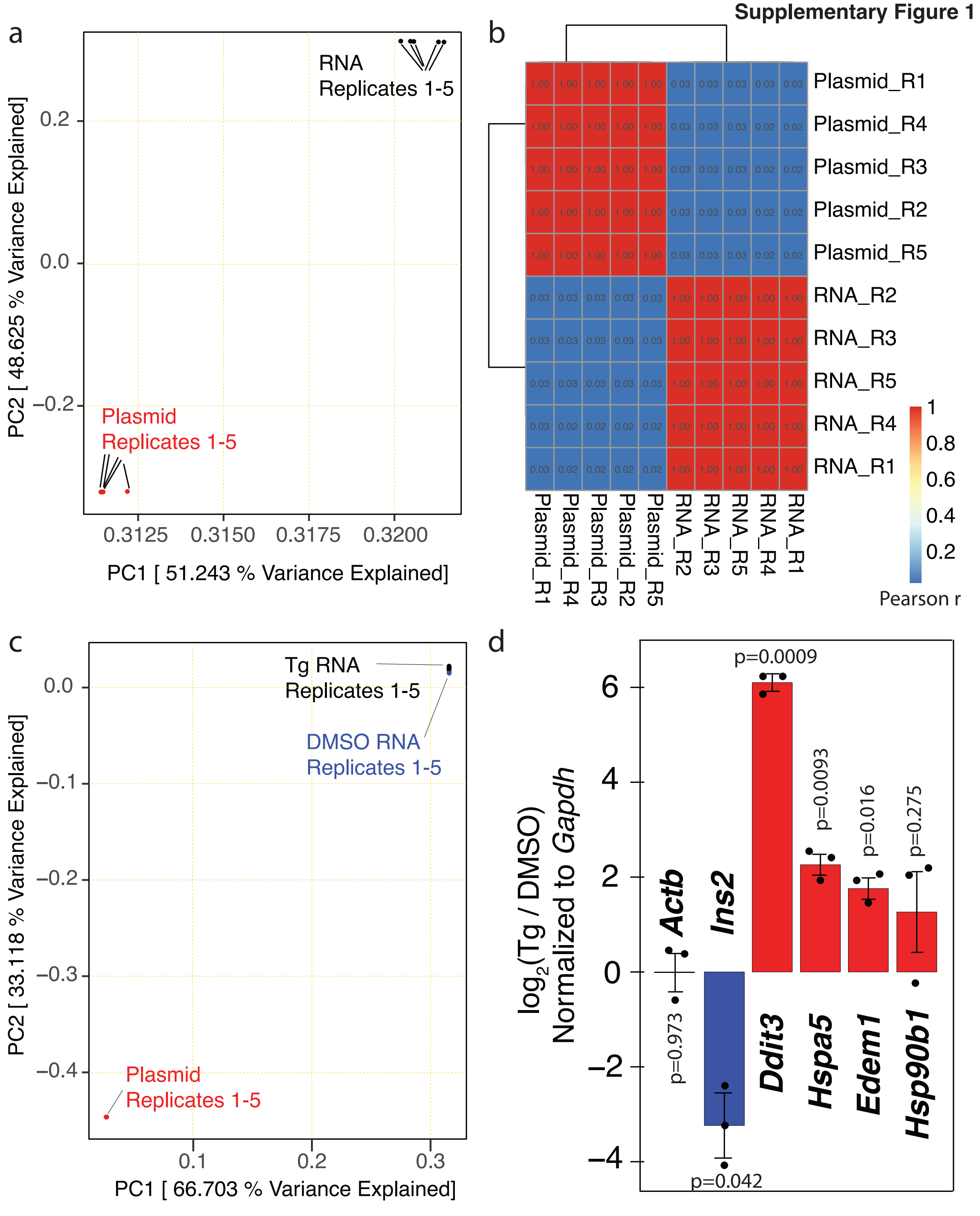

Supplementary Figure 1. **QC Metrics of MPRA libraries. (a)** Scatter plot of the first two principal components, which together explain >99% of the variation in 5 plasmid and 5 mRNA MPRA sequencing replicates. mRNA replicates were obtained after transfection of the MPRA library into MIN6 cells under standard culture conditions. **(b)** Heatmap of pairwise Pearson correlation coefficients with unsupervised row and column clustering. R1-5 denote the biological replicates. **(c)** Scatter plot of the first two principal components, which together explain >99% of the variation in five plasmid and ten mRNA MPRA sequencing replicates (five DMSO + five Tg). **(d)** RT-qPCR of ER stress response genes (*Ddit3, Hspa5, Edem1,* and *Hsp90b1*), *Ins2,* and *Actb* negative control gene in MIN6 cells treated with 250 nM of the ER stress inducing agent thapsigargin (Tg) or dimethylsulfoxide (DMSO) solvent-alone control for 24 hours. Bar plots show mean ± SEM of three biological replicates. *p<0.05; ** p<0.01; *** p<0.001, two-sided paired t-test comparing transcript abundance in MIN6 cells treated with 250 nM Tg to that in cells treated with DMSO.

Supplementary Figure 2

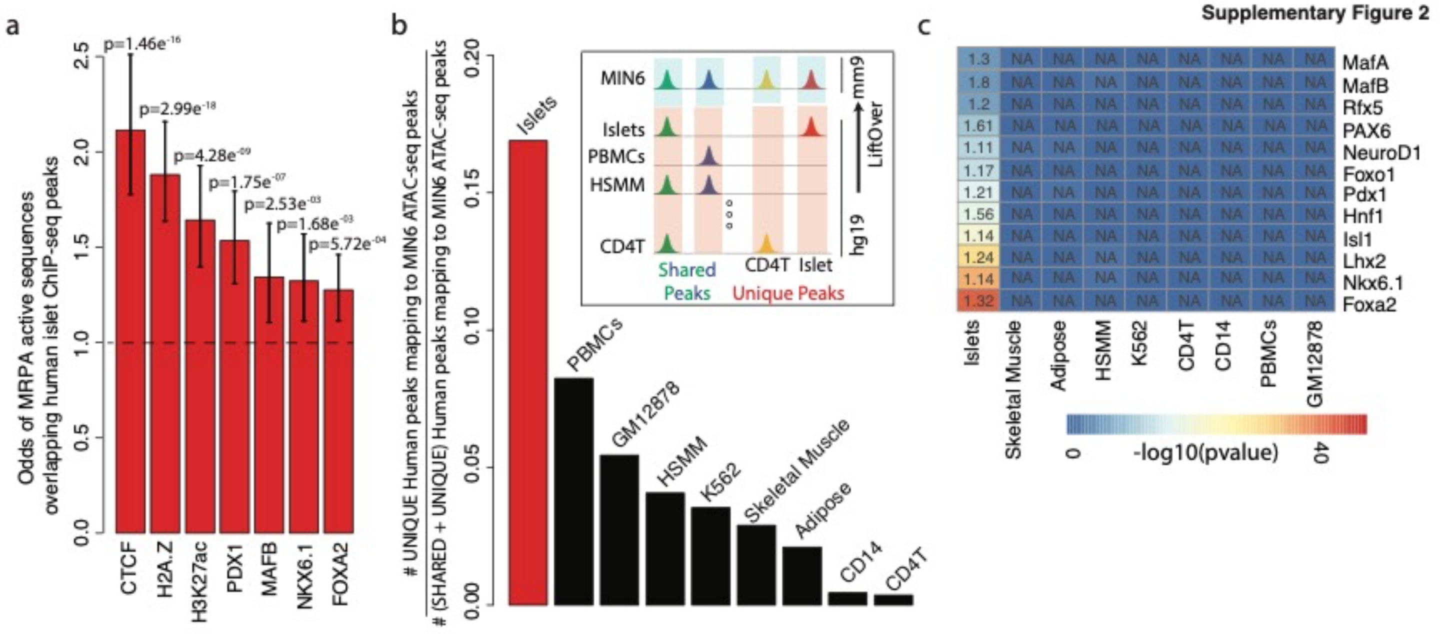

Supplementary Figure 2. **MIN6 mouse β cells are valid cell models to test human DNA sequences for β cell transcriptional activation potential. (a)** Bar plots displaying the odds of MPRA active elements overlapping *in vivo* human islet TF ChIP-seq[1] peaks compared to MPRA inactive elements. Error bars indicate 95% confidence intervals of the odds ratio estimates. ***p<0.001; **p<0.01, two-sided Fisher's Exact Test. **(b)** Fraction of ATAC-seq peaks from respective human tissues overlapping MIN6 ATAC-seq peaks (y-axis) that were unique to each of 9 human tissues or cell types analyzed (x-axis). ATAC-seq peaks from each human cell type were mapped to the mouse genome (mm9) using the UCSC Genome Browser liftover tool and overlapped with MIN6 ATAC-seq peak locations. **(c)** Heatmap of the enrichment of islet-specific TF motifs at human ATAC-seq peaks uniquely overlapping MIN6 ATAC-seq peaks. Colors in the heatmap represent -log10 (raw p-values) of significance calculated by HOMER using a hypergeometric test. Numbers indicate fold change of enrichment (%Target / %Background).

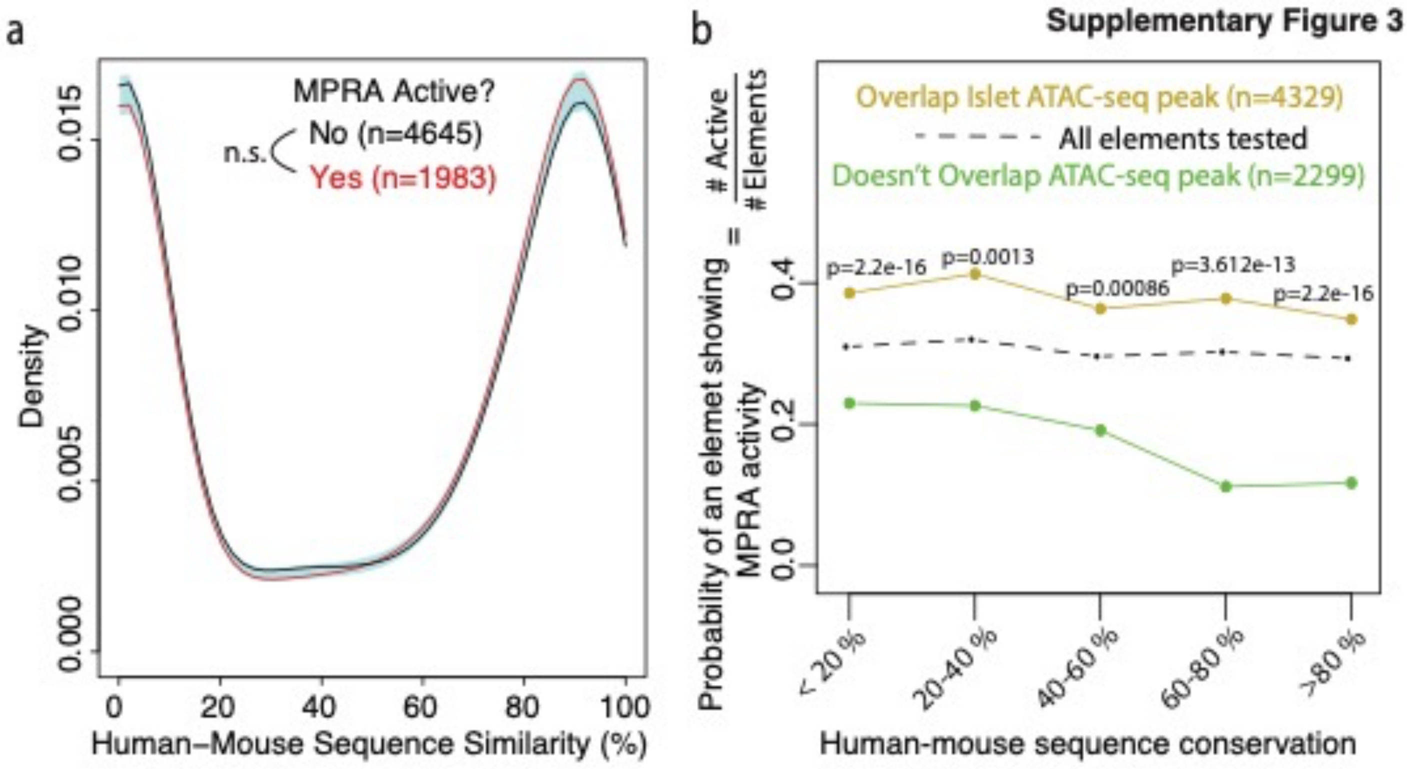

Supplementary Figure 3. **Human-mouse sequence similarity of MPRA active elements. (a)** Human-mouse sequence similarity for elements with (red) or without (black) significant MPRA activity. Human sequences that did not liftover to mm9 mouse genome build with ≤ 1% sequence similarity were classified as having 0% sequence similarity. A bootstrap hypothesis test of equality did not find a statistically significant difference between elements with (red) or without (black) MPRA activity for human-mouse sequence similarity. The upper and lower endpoints for equality are indicated as a blue reference band. **(b)** The probability that an element has significant MPRA activity (y-axis; at least 1 allele; any experimental condition) is shown as a function of sequence similarity (x-axis) between human and mouse genomes (expected probability is the black dotted line). Elements overlapping islet ATAC-seq peaks (brown line) had a significantly higher probability of MPRA activity than elements that did not overlap ATAC-seq peaks (green line), regardless of human-mouse sequence similarity. ***, **, and * denote p<0.001, 0.01, 0.05, respectively, using Bonferroni-corrected Fisher's exact test.

Supplementary Figure 4

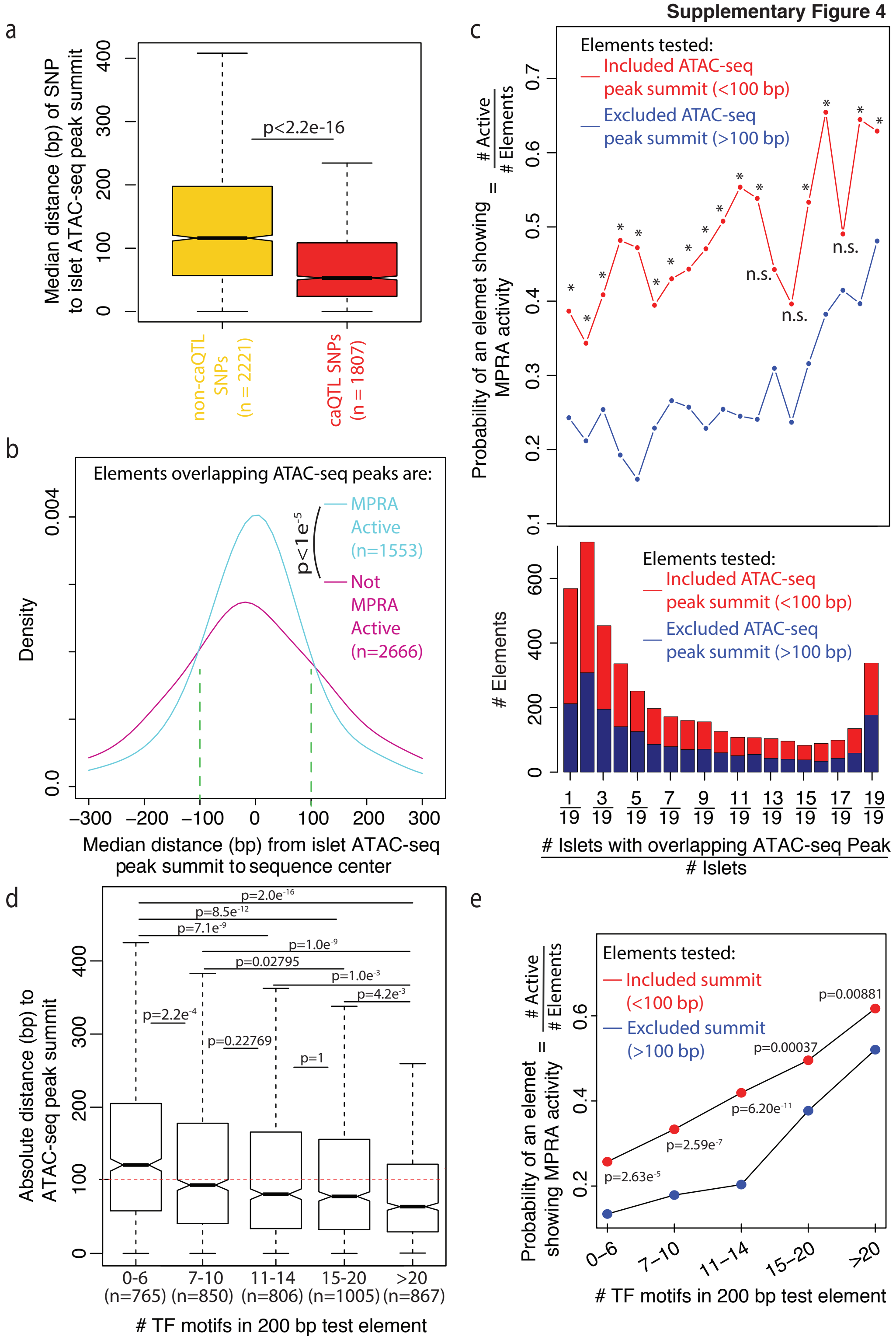

Supplementary Figure 4. MPRA activity is enriched at ATAC-seq peak summits. (a) caQTL SNPs (n=2,221) are closer to ATAC-seq peak summits than non-caQTL SNPs (n=1,807). P-value calculated using two-sided Wilcoxon test. **(b)** Elements in which SNPs centered within 200 bp sequences tested were ≤ 100 bp (green dashed lines) from ATAC-seq peak summits were more likely to be identified as MPRA active by performing a bootstrap analysis testing for equality between active and inactive sequences. This effect is restricted to SNPs within 100 bp of ATAC-seq peak summits because MPRA oligos were designed to test only 100 bp flanking SNPs of interest. ATAC-seq peak summits were therefore included in the sequences tested only if the SNP was within 100 bp of the ATAC-seq peak summit. **(c)** (Top) The probability of an element (≥1 allele) being identified as MPRA active (y-axis) binned by the number of islet donors (of 19 total) in whom the ATAC-seq peak was detected (x-axis). Elements where the ATAC-seq peak summit was included, i.e., SNP to ATAC-seq peak summit distance was <100 bp bps (red), were significantly more likely to be identified as MPRA active. *, FDR < 10%, two-sided Fisher's exact test. (Bottom) Stacked bar plot of the number of times an ATAC-seq peak overlapping the MPRA element is detected in the cohort (n = 19 islet donors). **(d)** SNP to ATAC-seq peak summit distances (y-axis) for tested 200 bp elements binned by the number of TF binding motifs they contain (x-axis). ***, **, and * denote p<0.001, 0.01, 0.05 using Bonferroni-corrected two-sided Wilcoxon test p-values, respectively. **(e)** 200 bp elements tested with MPRA are categorized based on the number of TF binding motifs detected and plotted against the probability of observing significant MPRA activity. Elements were grouped into those including (red) or excluding (blue) ATAC-seq summits. ***, p<0.001 using Bonferroni-corrected two-sided Fisher's Exact Test p-values. Box plots in panels **a** and **d** represent the minimum, maximum, median, first quartile and third quartile in the data set.

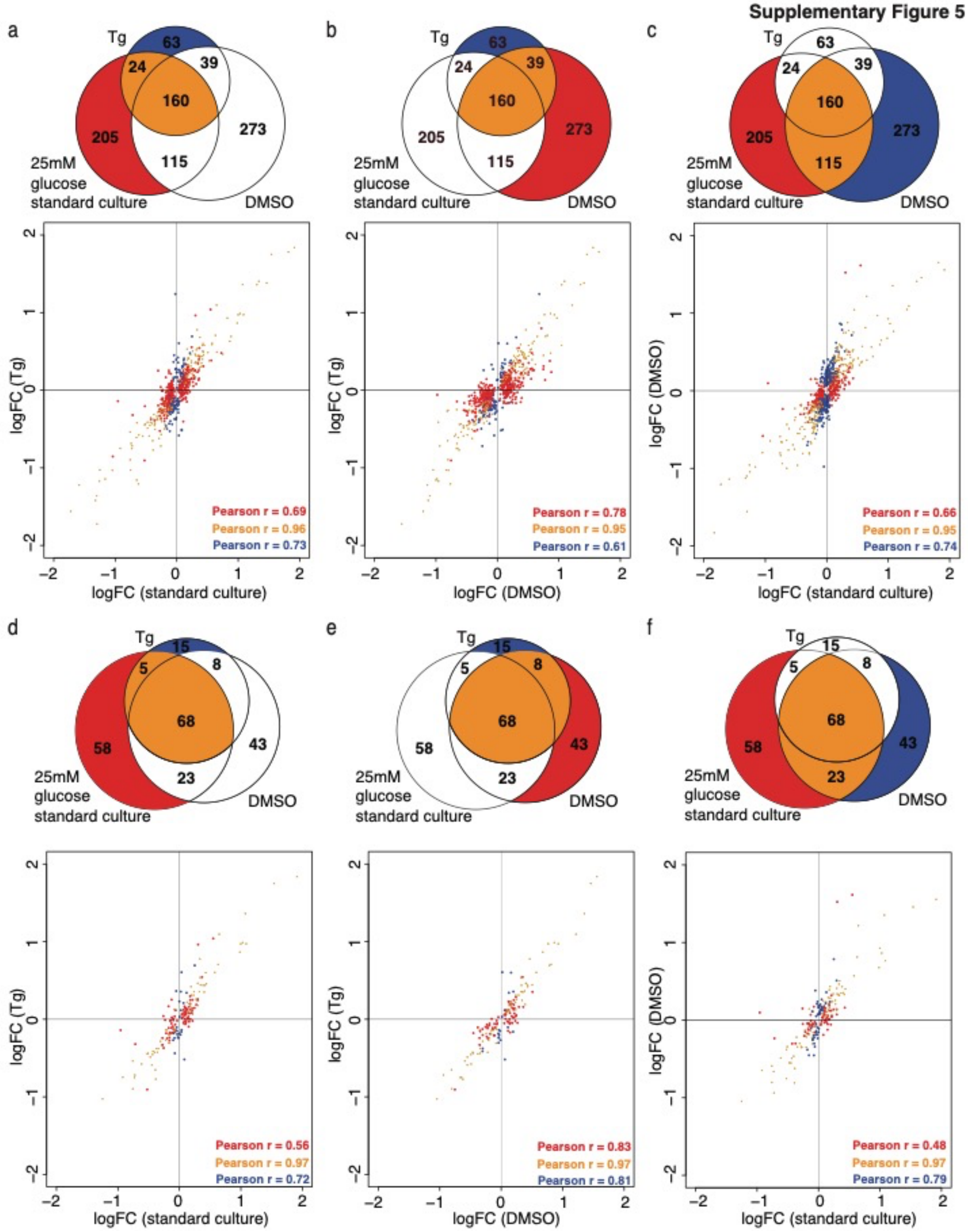

Supplementary Figure 5. **MPRA-altering effects of SNP alleles in standard (25mM glucose), thapsigargin, and/or DMSO culture conditions.** (a-c) Allelic effects on MPRA activity of 879 SNPs across the three tested conditions. (Top) Venn diagrams indicating the number of SNPs with significant (FDR<10%) allelic effects on MPRA activity for each condition. (Bottom) Scatter plots of $\log_2$ fold change in MPRA activity between each allele of SNPs with significant allelic effects in each condition tested. Points in the plot are color-coded to indicate SNPs with significant allelic effects in one (red, blue) or in both (orange) conditions compared according to the color scheme in the Venn diagram above. (d-f) Allelic effects on MPRA activity of 220 T2D-associated SNPs across the three tested conditions. Venn diagrams and scatter plot arrangement, designations, and color-coding are as indicated for panels a-c above. FC = fold change; Tg = thapsigargin.

Supplementary Figure 6

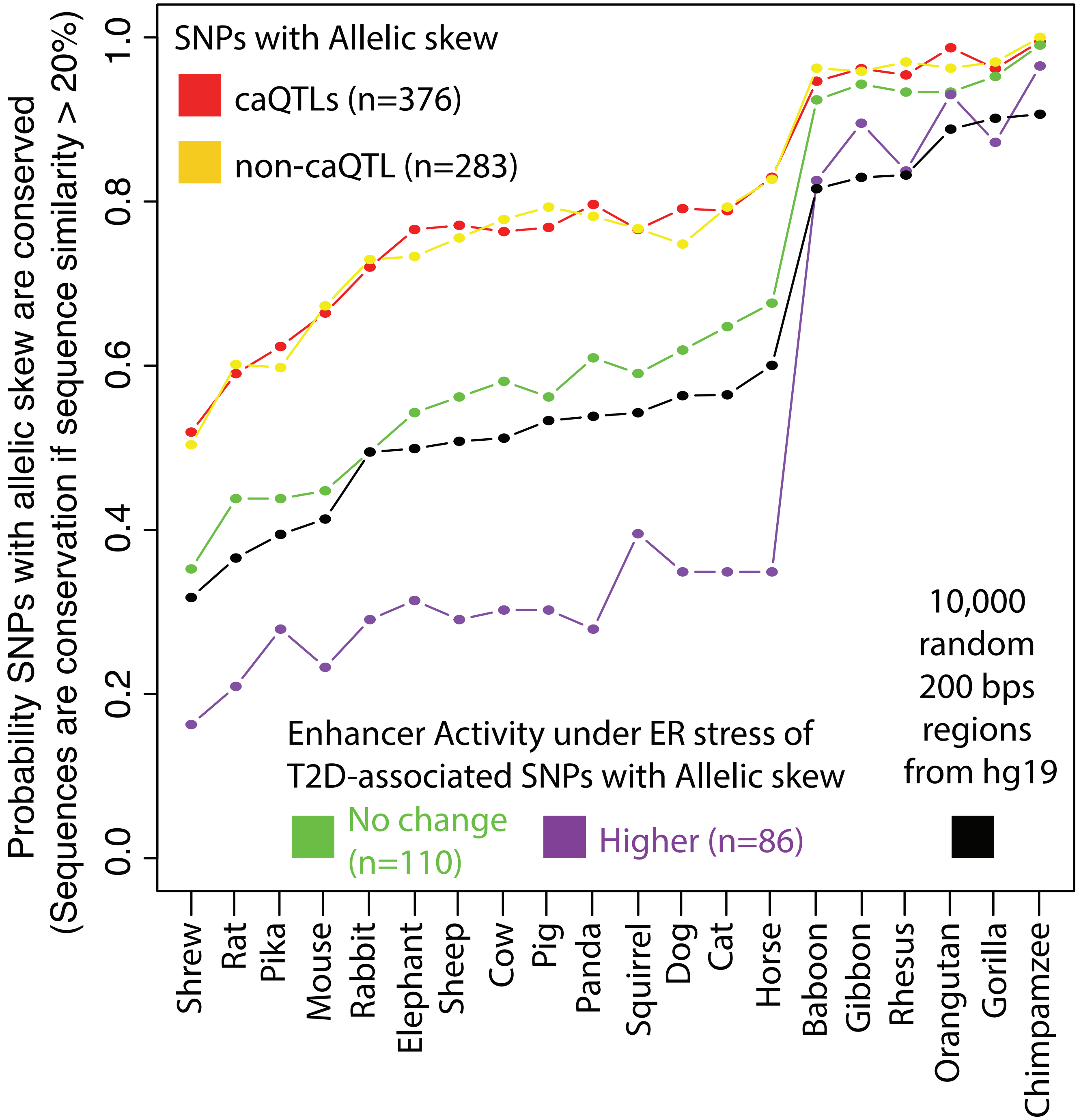

Supplementary Figure 6. **T2D SNP-containing elements with higher MPRA activity under ER stress are less likely to be conserved in non-primate mammalian species.** The probability that elements containing SNPs with allelic effects on MPRA activity are conserved with sequence similarity greater than 20% (y-axis) is plotted for 20 mammals whose genomes are available (x-axis). Red or yellow dots denote caQTL or non-caQTL SNPs, respectively. T2D SNP-containing elements with allelic effects were separated into two groups that either exhibited significantly higher MPRA activity under ER stress (purple dots; n=86) or no change (green dots; n=110). Compared to 10,000 random 200 bp regions from the human genome (black dots), elements overlapping caQTL and non-caQTL SNPs were significantly more likely to be conserved in non-primate mammalian species, while T2D SNP-containing elements with higher MPRA activity under ER stress were significantly less likely to be conserved in non-primate mammalian species (FDR<5%, Fisher's exact test).

References cited in Supplementary Figures:

1. Pasquali, L. *et al.* Pancreatic islet enhancer clusters enriched in type 2 diabetes risk-associated variants. *Nat. Genet.* **46**, 136–143 (2014).
