# Supplementary material for: Functional characterization of T2D-associated SNP effects on baseline and ER stress-responsive β cell transcriptional activation
Source: Nat Commun. 2021 Sep 2;12:5242. doi: 10.1038/s41467-021-25514-6 (PMC8413311; doi:10.1038/s41467-021-25514-6)
Supplement: Supplementary file 2 — Description of Additional Supplementary Files [file 41467_2021_25514_MOESM2_ESM.pdf]

Description of Additional Supplementary Files

File Name: Supplementary Data 1
Description: Transcription factor (TF) motifs enriched in MPRA active sequences under standard MIN6 culture conditions (25 mM glucose)

File Name: Supplementary Data 2
Description: Transcription factor (TF) motifs enriched in sequences with lower or higher activity in thapsigargin-treated (ER stress) vs. DMSO solvent control conditions

File Name: Supplementary Data 3
Description: MPRA allelic read counts of all SNPs (n=6621) tested in each of the three conditions (baseline, DMSO, thapsigargin)

File Name: Supplementary Data 4
Description: Transcription factor (TF) binding motifs predicted by MotifBreakR or SNP2TFBS to be disrupted by all SNPs exhibiting allelic MPRA activity.

File Name: Supplementary Data 5
Description: MPRA activity of each T2D-associated SNP allele tested in baseline, DMSO, or thapsigargin (ER stress) conditions

File Name: Supplementary Data 6
Description: T2D SNPs exhibiting allelic effects on MPRA activity. Each worksheet lists the SNP(s) in loci exhibiting one, two, or three functional SNPs altering MPRA activity in MIN6.

File Name: Supplementary Data 7
Description: T2D and caQTL SNP motif disruption predictions. Transcription factor (TF) motifs predicted by MotifBreakR or SNP2TFBS to be disrupted by T2D or caQTL SNPs exhibiting allelic effects on MIN6 MPRA activity

File Name: Supplementary Data 8
Description: Primers used in this study. Primer designations and sequences used for MPRA library preparation, quantitative (q)PCR, and electrophoretic mobility shift assay (EMSA) experiments.
