# Supplementary material for: Functional characterization of T2D-associated SNP effects on baseline and ER stress-responsive β cell transcriptional activation
Source: Nat Commun. 2021 Sep 2;12:5242. doi: 10.1038/s41467-021-25514-6 (PMC8413311; doi:10.1038/s41467-021-25514-6)
Supplement: Supplementary file 11 — Reporting Summary [file 41467_2021_25514_MOESM11_ESM.pdf]

nature research

Corresponding author(s): Michael L Stitzel, Ryan Tewhey

Last updated by author(s): 7/09/2021

Reporting Summary

Nature Research wishes to improve the reproducibility of the work that we publish. This form provides structure for consistency and transparency in reporting. For further information on Nature Research policies, see Authors & Referees and the Editorial Policy Checklist .

Statistics

For all statistical analyses, confirm that the following items are present in the figure legend, table legend, main text, or Methods section.

n/a	Confirmed	
☒	☐	The exact sample size (n) for each experimental group/condition, given as a discrete number and unit of measurement
☐	☒	A statement on whether measurements were taken from distinct samples or whether the same sample was measured repeatedly
☐	☒	The statistical test(s) used AND whether they are one- or two-sided<br>*Only common tests should be described solely by name; describe more complex techniques in the Methods section.*
☒	☐	A description of all covariates tested
☐	☒	A description of any assumptions or corrections, such as tests of normality and adjustment for multiple comparisons
☐	☒	A full description of the statistical parameters including central tendency (e.g. means) or other basic estimates (e.g. regression coefficient) AND variation (e.g. standard deviation) or associated estimates of uncertainty (e.g. confidence intervals)
☐	☒	For null hypothesis testing, the test statistic (e.g. F, t, r) with confidence intervals, effect sizes, degrees of freedom and P value noted<br>*Give P values as exact values whenever suitable.*
☒	☐	For Bayesian analysis, information on the choice of priors and Markov chain Monte Carlo settings
☒	☐	For hierarchical and complex designs, identification of the appropriate level for tests and full reporting of outcomes
☐	☒	Estimates of effect sizes (e.g. Cohen's d, Pearson's r), indicating how they were calculated

Our web collection on statistics for biologists contains articles on many of the points above.

Software and code

Policy information about availability of computer code

Data collection	Illumina sequencing data was collected from MIN6 samples according to details in the Methods section.
Data analysis	Code for analyses completed in this study are available at https://github.com/UcarLab/MPRA_Khetan and at Zenodo link https://doi.org/10.5281/zenodo.4974390

For manuscripts utilizing custom algorithms or software that are central to the research but not yet described in published literature, software must be made available to editors/reviewers. We strongly encourage code deposition in a community repository (e.g. GitHub). See the Nature Research guidelines for submitting code & software for further information.

Data

Policy information about availability of data

All manuscripts must include a data availability statement. This statement should provide the following information, where applicable:

- Accession codes, unique identifiers, or web links for publicly available datasets
- A list of figures that have associated raw data
- A description of any restrictions on data availability

All data generated in this study is made publicly available on GEO. Accession for the data is GSE145643.

Field-specific reporting

Please select the one below that is the best fit for your research. If you are not sure, read the appropriate sections before making your selection.

☒ Life sciences ☐ Behavioural & social sciences ☐ Ecological, evolutionary & environmental sciences

Life sciences study design

All studies must disclose on these points even when the disclosure is negative.

Sample size	Five biological replicates were completed for each condition based on the MPRA protocol in Tewhey et al., 2016 (Cell), which established sensitivity and specificity parameters for identifying active sequences and variants with allelic imbalances (skew) associated with eQTLs.
Data exclusions	No data was excluded from analysis.
Replication	Five biological replicates (different batches of cells, different days, and different transfections) were performed for each MPRA experiment to ensure reproducibility. Principal component analyses indicated significant correlation between biological replicates. Allelic imbalances (skew) also exhibited significant positive correlation across experiments and conditions.
Randomization	Each replicate sample was obtained from cells transfected with a plasmid pool of thousands of elements and hundreds of barcodes (millions of unique plasmid sequences). Simultaneous transfection of this library pool into a population of millions of cells effectively served as randomization.
Blinding	Blinding is not applicable. Biological replicates clustered according to condition based on their RNA-seq similarities. For each condition, cells were transfected with a plasmid library pool of thousands of sequence elements, each with hundreds of barcodes, and activity of these sequences was determined in an agnostic manner using RNA-seq as the output.

Reporting for specific materials, systems and methods

We require information from authors about some types of materials, experimental systems and methods used in many studies. Here, indicate whether each material, system or method listed is relevant to your study. If you are not sure if a list item applies to your research, read the appropriate section before selecting a response.

Materials & experimental systems

n/a	Involved in the study
☒	☐ Antibodies
☐	☒ Eukaryotic cell lines
☒	☐ Palaeontology
☒	☐ Animals and other organisms
☒	☐ Human research participants
☒	☐ Clinical data

Methods

n/a	Involved in the study
☒	☐ ChIP-seq
☒	☐ Flow cytometry
☒	☐ MRI-based neuroimaging

Eukaryotic cell lines

Policy information about cell lines

Cell line source(s)	MIN6 beta cell line used was originally derived by Miyazaki et al., (1990)
Authentication	Identity of MIN6 as murine beta cell line was authenticated by parallel insulin content, ATAC-seq, RT-qPCR, and RNA-seq experiments/analyses during the study period.
Mycoplasma contamination	MIN6 was tested for mycoplasma and found negative prior to expansion and creation of cryopreserved stocks in the lab.
Commonly misidentified lines (See ICLAC register)	No commonly misidentified lines were used during this study.
